# The role of phages for microdiverse bacterial communities in proglacial stream biofilms

**DOI:** 10.3389/frmbi.2023.1279550

**Published:** 2024-01-15

**Authors:** Hannes Peter, Grégoire Michoud, Susheel Bhanu Busi, Tom J. Battin

**Affiliations:** ^1^ River Ecosystems Laboratory, Alpine and Polar Environmental Research Center, Ecole Polytechnique Fédérale de Lausanne, Sion, Switzerland; ^2^ Systems Ecology Group, Luxembourg Centre for Systems Biomedicine, University of Luxembourg, Esch-sur-Alzette, Luxembourg; ^3^ UK Centre for Ecology & Hydrology (UKCEH), Wallingford, Oxfordshire, United Kingdom

**Keywords:** phage-host interactions, host range, auxiliary metabolic genes, environmental selection, coevolution

## Abstract

Viruses modulate the diversity and activity of microbial communities. However, little is known about their role for the structure of stream bacterial biofilm communities. Here, we present insights into the diversity and composition of viral communities in various streams draining three proglacial floodplains in Switzerland. Proglacial streams are characterized by extreme environmental conditions, including near-freezing temperatures and ultra-oligotrophy. These conditions select for few but well-adapted bacterial clades, which dominate biofilm communities and occupy niches via microdiversification. We used metagenomic sequencing to reveal a diverse biofilm viral assemblage in these streams. Across the different floodplains and streams, viral community composition was tightly coupled to that of the bacterial hosts, which was underscored by generally high host specificity. Combining predictions of phage-host interactions with auxiliary metabolic genes (AMGs), we identify specific AMGs shared by phages infecting microdiverse clade members. Our work provides a step towards a better understanding of the complex interactions among bacteria and phages in stream biofilm communities in general and streams influenced by glacier meltwaters and characterized by microdiversity in particular.

## Introduction

Viruses infecting bacteria, also called bacteriophages, play important roles in modulating the diversity and activity of bacterial assemblages in every biome on Earth (Dion et al., 2020). Particularly phage-induced mortality is known to control bacterial abundance and biomass, community composition and diversity (Suttle, 1994; Suttle, 2007; Roux et al., 2016). Metabolic reprogramming during lytic infections redirects host metabolism towards viral progeny production, with sizable impacts on ecosystem-scale biogeochemical cycles (reviewed by: Zimmerman et al., 2020). However, beyond altering host’ metabolic pathways and inducing mortality, phages influence the fitness of their bacterial hosts in numerous ways. Lysogenic infections, for example, can impede host functioning by insertion of phage genetic material into functional genes (reviewed by: Obeng et al., 2016). And phage-encoded sigma factors can influence bacterial spore formation, affecting important bacterial life-history traits such as dormancy (Schwartz et al., 2022).

While the consequences of viral infections for individual bacterial cells are often detrimental, beneficial effects of phage-host interactions typically arise at the level of populations, communities or even holobionts (Shkoporov et al., 2022). For instance, phage-driven diversification (Koskella and Brockhurst, 2014), lysogenic conversion (Howard-Varona et al., 2017) or auxiliary metabolic genes [AMGs (Kieft et al., 2021b)] can increase phenotypic plasticity or augment the metabolic repertoire of microbial assemblages (reviewed by: Shkoporov et al., 2022). In biofilms, where densely packed communities of microbes interact, phage genomic material has been shown to enter even phylogenetically distant hosts directly or during conjugative transfers, ultimately increasing the immunological memory (Hwang et al., 2023).

Besides the density, diversity, and activity of phages and their hosts, the eco-evolutionary consequences of phage infections depend on host specificity. While a notion of a predominantly narrow phage host range prevailed in early work, recent advances, including single-cell and metagenomics studies of hydrothermal biofilms (Jarett et al., 2020; Hwang et al., 2023), suggest indeed a continuum of host ranges (reviewed by: de Jonge et al., 2019; Holtappels et al., 2023).

Here, we present insights into the interactions between bacterial biofilm communities and their phages in high-altitude stream ecosystems. Alpine streams are extreme environments, characterized by ultra-oligotrophy, low water temperature, exposure to ultraviolet (UV) radiation, and short ice-free seasons, yet represent hotspots of microbial biodiversity (Freimann et al., 2013; reviewed by: Hotaling et al., 2017; Brandani et al., 2022; Busi et al., 2022; Fodelianakis et al., 2022). Glacier influence adds additional constraints for life in alpine streams. Close to the glacier terminus, glacier-fed streams (GFS) are permanently ice cold and the erosive activity of glaciers liberates large quantities of mineral particles, which render GFS turbid and limit light for phototrophic primary production. Compared to GFS, groundwater-fed streams (GWS) that drain elevated terrain towards the edges of the floodplains, are warmer, their streambeds more consolidated and lower turbidity allows photosynthetic primary producers to dwell (Freimann et al., 2013; Brandani et al., 2022).

Previous work on biofilm communities in GFS and GWS revealed that they are diverse with members of all domains of life present (reviewed by: Battin et al., 2016; Brandani et al., 2022), distinct from the microbial community suspended in the water column (Ezzat et al., 2022), and shaped predominantly by environmental selection (Fodelianakis et al., 2022; Brandani et al., 2023). More specifically, this means that ecologically successful taxa in both GFS and GWS are recruited from similar clades, including taxa classified as *Polaromonas*, *Rhodoferax*, *Rhizobacter*, *Methylotenera*, and *Massilia*. These genera are microdiverse (Fodelianakis et al., 2022), indicating that they efficiently fill available niches which cannot be occupied by less-well adapted taxa. Microdiversity is an intrinsic property of many microbial communities (reviewed by: Larkin and Martiny, 2017), including marine viral communities (Needham et al., 2017; Gregory et al., 2019).

By focusing on microdiverse biofilm communities in proglacial streams, we aim to shed new light on the role of phage-host interactions in microbial communities in extreme environments. We hypothesize that narrow phage host ranges predominate in communities dominated by microdiverse clades because selective constraints which lead to microdiversification may also shape bacteria-phage coevolution. Moreover, we speculate that interactions between phages and microdiverse clade members in extreme environments may be predominantly beneficial, thus contributing to the ecological success of microdiverse clades in extreme environments. The rationale behind this is that reciprocal feedbacks between beneficial effects of phage-host interactions and the density of hosts could stabilize the process of microdiversification over evolutionary timescales – in contrast to a situation where density-dependent phage predation would lead to turnover in host communities (and thus suppress microdiversification). We argue that studying microbial communities in alpine stream biofilms is ideally suited to address such questions, because of the pronounced microdiversity and limited dispersal due to geographic and topographic isolation, which can increase phage-host encounter probabilities and hence lessen the coevolutionary strength of phage-host associations.

## Materials and methods

### Study sites

We here present data from an extensive survey of benthic biofilm communities sampled from proglacial streams in the Swiss Alps in summer 2019. Detailed descriptions of the sampling design and environmental parameters as well as analyses of spatial diversity patterns (Brandani et al., 2022), assembly processes (Brandani et al., 2023) and the functional diversity (Michoud et al., 2023) are available elsewhere. In this field survey, a total of 259 benthic sediment samples were collected from the Otemma Glacier (OTE, 45.95E, 7.45N), the Valsorey Glacier (SOY, 45.91E, 7.27N) and Val Roseg Glacier (VAR, 46.39E, 9.84N) floodplains. The sampling sites were distributed along gradients from the glacier snout to the outflows of the respective proglacial floodplains, covering terrain which has been deglaciated since ca. 36 (OTE), 65 (VAR), and 70 (SOY) years. Samples of the coarse sandy sediment fraction were collected using flame-sterilized shovels (0-5 cm depth) and fractionated using flame-sterilized sieves (Retsch, 0.25 and 3.15 mm). Subsamples were immediately flash-frozen on dry ice and stored at -80°C until processing. Samples for bacterial cell counting were fixed using 1.8 mL of a mixture containing filter-sterilized paraformaldehyde (1% w/v) and 0.5% (w/w) glutaraldehyde, flash frozen and kept at -80°C (Brandani et al., 2022; Fodelianakis et al., 2022). After extraction from sediments using pyrophosphate, shaking and sonication, bacterial cells were stained using SYBR Green I (1 x final concentration; Thermo Fisher) for 15 minutes at 37°C and counted using flow cytometry (NovoCyte, ACEA) equipped with a 488 nm laser. Samples were analyzed with a reading time of 2 minutes at a flow rate of 14 µL min^-1^ and thresholds on the forward scatter channel set to 300 and the 530/30 nm fluorescence channel set to 1500. Cells were distinguished from background noise based on fluorescence signals on biplots of 530/30 and 725/40 nm using the NovoExpress (ACEA) software. Cell abundances were converted to cells per g dry sediment.

Here, we focus on the viral communities, which we assess using bulk metagenomic sequencing of a subset of samples. We obtained metagenomes for 47 samples from GFS (n=12) and GWS (n=35) from OTE (n_GFS_=3, n_GWS_=14), SOY (n_GFS_=6, n_GWS_=10) and VAR (n_GFS_=3, n_GWS_=11), respectively. Twenty-four of these samples were obtained in June–July, and 23 samples were collected in August–September 2019 (**Supplementary Table S1**).

### DNA extraction, library preparation, and sequencing

DNA was extracted from 0.5 g of sediment using an optimized extraction protocol tailored to the low-biomass and mineral nature of these samples (Busi et al., 2020). Metabarcoding libraries were prepared using 2-3 ng µL^-1^ input DNA and primers targeting the V3-V4 hypervariable region of the 16S rRNA gene [341f (5’-CCTACGGGNGGCWGCAG-3’) and 785r (5’-GACTACHVGGGTATCTAATCC-3’) (Klindworth et al., 2013)]. Amplification was performed on a Biometra Trio (Biometra) using the KAPA HiFi DNA Polymerase (Hot Start and Ready Mix formulation) in a 25 μL-amplification reaction containing 1 x PCR buffer, 1 μM of each primer, 0.48 μg μL^−1^ bovine serum albumin (BSA), and 1.0 μL of template DNA. After an initial denaturation step at 95°C for 3min, 25 cycles of 94°C for 30 s, 55°C for 30 s, and 72°C for 30 s were followed by a final extension at 72°C for 5 min. Sequencing libraries were prepared using dual indices (Illumina). Prior to paired-end sequencing on a single MiSeq (Illumina) lane, library DNA concentrations were quantified, normalized, and pooled. Libraries were then sequenced at the Lausanne Genomic Technologies Facility (Switzerland), producing 300 bp paired end reads.

Metagenome libraries were prepared using the NEBNext Ultra II FS library kit, with 50 ng of input DNA. Briefly, DNA was enzymatically fragmented for 12.5 min and amplified using 6 PCR cycles. Qubit (Invitrogen) and Bioanalyzer (Agilent) were used to check the quality and insert size of the libraries (450 bp). The metagenomes were sequenced at the Functional Genomics Centre Zürich using an S4 flowcell on a NovaSeq (Illumina) platform, producing 150 bp paired end reads.

### Bioinformatic processing

Amplicon sequences were quality checked using Trimmomatic v0.36 (Bolger et al., 2014) and assigned to exact Amplicon Sequence Variants (ASVs) using DADA2 v1.10.0 (Callahan et al., 2016) implemented in QIIME2 v2020.8 (Bolyen et al., 2019) with default parameters. Singleton ASVs were removed and taxonomy was assigned using the naïve Bayesian classifier implemented in QIIME2 and the SILVA v138.1 reference database. Non-bacterial ASVs (i.e. chloroplasts, mitochondria, and archaea) were discarded. We built *de novo* a phylogenetic tree using RAxML v8.2.12 (Stamatakis, 2014) and the GTRCAT substitution model and the rapid bootstrapping option. Phylogenetic distances among ASVs were calculated using function *cophenetic* implemented in R v4.1 (R Core Team, 2021).

Metagenomic sequence reads were preprocessed using trim_galore v0.6.6 (Krueger et al., 2021), which uses fastqc v0.11.9 (Andrews, 2010) for quality control and cutadapt v3.4 (Martin, 2011) for adapter removal. Trimmed reads were assembled separately for each sample with megahit v1.2.9 (Li et al., 2015) using default parameters and a minimum contig length of 1,000 bp. Prokaryotic Metagenome-Assembled Genomes (MAGs) were binned using MetaBAT2 v2.15 (Kang et al., 2019) and CONCOCT v1.1 (Alneberg et al., 2014). Reads were mapped to the assemblies with CoverM v0.6.1 (https://github.com/wwood/CoverM). DAS Tool v1.1.2 (Sieber et al., 2018) was used to obtain a non-redundant set of MAGs for each sample. All MAGs were then dereplicated with dRep v3.2.2 (Olm et al., 2017), considering a completeness level greater than 75% and a contamination level less than 10% as determined by checkM v1.1.3 (Parks et al., 2015). Taxonomy of MAGs was assigned using GTDB-Tk v1.7.0 (Chaumeil et al., 2019) based on release 202 obtained from https://data.ace.uq.edu.au/public/gtdb/data/releases/ (Quast et al., 2013). DefenseFinder v1.0.9 (Tesson et al., 2022) using default settings was used to identify antiviral systems on MAGs.

Putative viral contigs (n=57168) were identified from the assemblies using VIBRANT v1.2.1 (Kieft et al., 2020) with default parameters. VIBRANT also provides information about auxiliary metabolic genes on viral contigs based on KEGG annotations (i.e. including “metabolic pathways” and “sulfur relay system”). Complete viral contigs were then identified using CheckV v0.8.1 (Nayfach et al., 2021) and set aside (n=801). The remaining, non-complete viral contigs were binned into viral metagenome-assembled genomes (vMAGs) with PHAMB v1.0.1 (Johansen et al., 2022) which uses VAMB v3.0.2 (Nissen et al., 2021) and DeepVirFinder v1.0 (Ren et al., 2020) to obtain high-quality viral genomes. Subsequently, complete viral genomes and high-quality vMAGs were dereplicated using vRhyme v1.1.0 (Kieft et al., 2022) taking the longest bin and 97% identity to obtain a final set of vMAGs. CheckV was then used to assess vMAG completeness and quality. To obtain vMAG abundances, reads were mapped to the concatenated vMAG sequences using CoverM v0.6.1 in contig mode, using 100% minimum read identity, and the “trimmed_mean” method, which removes 5% of the highest and lowest coverages, respectively. The number of reads mapped to vMAGs are reported in **Supplementary Table S1**. An additional detection threshold of 5% of the contig length covered was applied and vMAG abundances below this threshold were set to zero. Coverage was then normalized (i.e. to range between 0 and 1) and treated as relative abundances in downstream analyses. BACPHLIP v0.9.6 (Hockenberry and Wilke, 2021) was used to assign temperate or virulent lifestyles to complete vMAGs. Viral taxonomy was assigned using kaiju v1.9.0 (Menzel et al., 2016) and the *viruses* database (only viruses from the NCBI RefSeq database, accessed 07/05/2022). PHIST v1.1.0 (Zielezinski et al., 2022), an alignment-free, k-mer frequency-based tool was used to predict phage-host interactions (based on p_adjusted_ <0.05). A snakemake workflow for our bioinformatic analyses can be found here: https://github.com/hpeter0803/viromes/tree/ensemble_paper.

### Statistical analyses

The statistical software language R v4.1 was used to prepare figures and to perform all statistical analyses. Specifically, viral taxonomic composition was visualized using the heat_tree function of the *metacoder* v*0.3.6* (Foster et al., 2017) R package. Differential abundance analysis for vMAGs was performed using DESeq2 v3.15 (Love et al., 2014). Multivariate analyses of bacterial (16S rRNA gene amplicon) and viral (vMAG) community composition included non-metric multidimensional scaling ordination (*metaMDS*), procrustes correlation (*procrustes* and *protest*), and permutational multivariate analysis of variance using distance matrices (PERMANOVA, *adonis2*) using Bray-Curtis dissimilarities and were performed using the R package *vegan v 2.6-4* (Oksanen et al., 2022) with default settings. Prior to these analyses, Wisconsin double standardization was applied to relative abundances and AMG counts were Hellinger transformed prior to Principal Component Analysis. Phage-host interactions were visualized using Cytoscape v3.10.0 (Shannon et al., 2003).

## Results and discussion

### Bacterial communities in proglacial streams are characterized by microdiversity

After removal of singletons, we retained 35,170 bacterial ASVs across the three different floodplains and the different stream types. The bacterial communities were taxonomically dominated by members of Gamma- and Alphaproteobacteria (17.4 and 8.2% of ASVs, respectively), Bacteroidia (12.5%), Planctomycetes (7.8%), and Verrucomicrobiae (6.4%). This diversity was further classified into 925 different genera, of which members of *Candidatus Nomurabacteria* (1.8% of ASVs), *Flavobacterium* (1.7%), and *Candidatus Saccharimonadales* (1.4%) accounted for most ASV richness.

**Figure 1:**
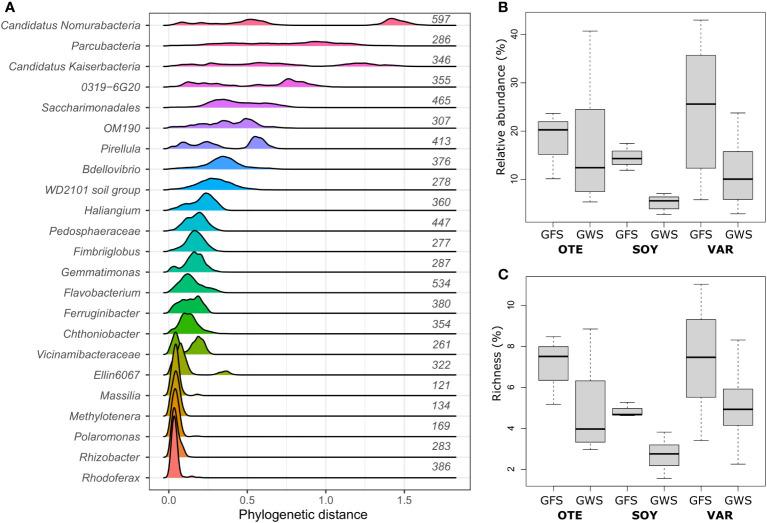
Microdiverse bacterial clades. Microdiverse bacterial genera in proglacial streams are abundant, prevalent and characterized by short pairwise phylogenetic distances among ASVs. Shown are the distributions of phylogenetic distances among ASVs with same genus-level taxonomy for the most abundant genera **(A)**. Note that e.g. *Parcubacteria*, *Saccharimonadales, Pedosphaeraceae*, and Vicinamibacteraceae refer to genus-level taxonomic assignments of further unclassified ASVs. The number of ASVs per genus is given to the right of each distribution. Microdiverse genera, i.e. *Rhodoferax*, *Rhizobacter*, *Polaromonas*, *Methylotenera*, and *Massilia*, were particularly abundant in GFS **(B)** while they contributed disproportionately less to overall community richness **(C)**.

Previous work has identified environmental factors common to both GFS and GWS, such as low water temperature (average 6.5 ± 4.6°C) and ultra-oligotrophy to shape proglacial stream biofilm communities (Brandani et al., 2022). Yet, important environmental differences between GFS and GWS, such as turbidity and diurnal variation in stream flow select for different ASVs which account for the compositional (Freimann et al., 2013; Brandani et al., 2022; Brandani et al., 2023) and functional differences (Michoud et al., 2023) between stream types. Leveraging phylogenetic and ecological signatures, we previously related environmental selection to microdiversity, particularly among members of the Gammaproteobacterial family *Comamonadacea* in proglacial streams in New Zealand (Fodelianakis et al., 2022) and the European Alps (Brandani et al., 2023). Here, we re-iterate this and use phylogenetic distances between ASVs, prevalence and relative abundance to identify microdiverse bacterial clades. We found that the genera *Polaromonas*, *Rhodoferax*, *Rhizobacter*, *Methylotenera*, and *Massilia* featured consistently short phylogenetic distances among ASVs (**Figure 1A**) and are microdiverse. Short phylogenetic distances could be a consequence of the number of ASVs with the same taxonomic classification. Indeed, we found that genera with few ASVs had generally shorter phylogenetic distances than genera containing many ASVs. *Polaromonas (n_ASVs_=169)*, *Rhodoferax (n_ASVs_=386)*, *Rhizobacter (n_ASVs_=283)*, *Methylotenera (n_ASVs_=134)*, and *Massilia (n_ASVs_=121)*, however, contain numerous ASVs, comparable to other, non-microdiverse bacterial genera (**Figure 1A**). And while these five microdiverse genera account on average ( ± SD) for only 5.0 ± 2.3% of ASV richness detected in each sample, they contribute 14.5 ± 11.3% to relative abundance (**Figures 1B, C**).

We obtained 2732 high-quality bacterial MAGs and previous work unraveled the functional differences between MAGs found in GFS and GWS (Michoud et al., 2023). Here, we used MAGs to identify phage-host interactions and screened them for their anti-phage arsenal. Nevertheless, MAGs, while only resolving a subset of the ASVs-resolved microbial diversity, reflected the composition of bacterial communities (**Supplementary Figure S1**). Specifically, matching ASVs and MAGs with same genus-level classification, mean relative abundance of MAGs was well correlated with relative abundance of ASVs (Spearman’s rho = 0.65, p<0.01).

### Viral communities in proglacial stream biofilms are diverse

**Figure 2:**
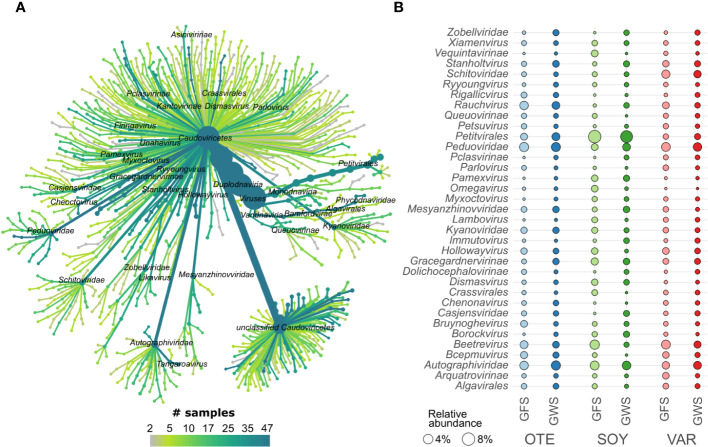
Viral taxonomic composition. vMAGs were classified mostly as members of *Caudoviricetes* **(A)**. Displayed is a tree-like representation of viral taxonomy. We found high prevalence (the number of samples in which a specific taxonomic group was detected) across several taxonomic groups. The mean relative abundance (circle size, panel **B)** of the most abundant vMAGs highlights differences across floodplains (e.g. *Autographiviridae, Peduoviridae*, and the eukaryotic viruses *Algalvirales*) and differences between GFS and GWS (e.g. *Petitvirales, Beetrevirus*, and *Kyanoviridae;* see **Supplementary Figure S2A** for more detail).

Generally, little is known about viral biodiversity in extreme environments, particularly in mountain streams and rivers (Payne et al., 2020; Bekliz et al., 2022; Busi et al., 2022). Here, we identified 1452 high-quality (n=870) or complete (n=582) vMAGs. vMAG genome size varied considerably, with complete vMAG genome size averaging 47.8 kbp (range 2.6 to 373.3 kbp) and high-quality vMAG size averaging 70.4 kbp (range: 5.0 to 622.1 kbp). Taxonomically, viral communities were dominated by viruses classified as *Caudoviricetes* (**Figure 2**). *Caudoviricetes* classified as *Ralstonia phage*, *Tsukubavirus*, and *Gordonia phage* were particularly abundant in GFS, whereas, unclassified viruses, *Unahavirus* and *Koutsourovirus* were relatively more abundant in GWS (**Supplementary Figure S2A**). Besides *Caudoviricetes* (n=930), *Malgrandaviricetes* (n=60), and *Megaviricetes* (n=6) constituted the viral communities.

On average, 225.4 ± 74.7 vMAGs were detected in each sample with no significant differences in viral richness across stream types (ANOVA, F=0.85, p=0.38) or floodplains (ANOVA, F=1.35, p= 0.26) (**Supplementary Figure S3**). Across the three floodplains, 831 vMAGs were present in GFS samples and 1350 vMAGs were present in GWS samples. While 15 vMAGs were present in all samples, 52 vMAGs were found in both stream types and across all three floodplains, forming the viral proglacial stream biofilm core community (**Supplementary Figure S2B**). These 52 core vMAGs accounted on average for 9.0% of vMAG relative abundance, with a notable overrepresentation in GFS of OTE, where they represented 33.5 ± 12.9% of vMAG relative abundance. A large number of vMAGs were exclusively found in GWS of SOY (n=264), VAR (n=150), and OTE (n=116) (**Supplementary Figure S2B**). In contrast, fewer vMAGs were exclusively present in GFS of VAR (n=45), SOY (n=31), and OTE (n=19).

Across all floodplains and stream types, most vMAGs were rare (i.e. low abundance) and only few vMAGs were abundant (i.e. reached more than 0.1% of relative abundance in any given sample), yet, these abundant vMAGs accounted on average for 96.8% of total viral relative abundance (**Supplementary Figures S3A, C**). Screening for lysogeny-associated protein domains (e.g. integrases and recombinases) on complete vMAG genomes (n=582), we found that 81.0% of them featured a virulent lifestyle, whereas 19.0% followed a temperate lifestyle (**Supplementary Figure S4**). Contrary to Killing-the-winner and Piggyback-the-winner model expectations (Thingstad and Lignell, 1997; Knowles et al., 2016; Silveira and Rohwer, 2016; reviewed by: Chen et al., 2021), we found no relationship between the ratio of temperate to virulent lifestyles (mean ratio: 0.41 ± 0.21) and bacterial abundance, despite a range of two orders of magnitude in bacterial abundance across our samples (**Supplementary Figure S4**). Additional work would be needed to better constrain these predictions (i.e. extending beyond complete vMAGs and accounting for novelty). Moreover, bulk metagenomics, as compared to viromics, may better capture actively replicating viruses but may offer only limited resolution to resolve non-infecting or rare viruses (i.e. viruses infecting rare hosts (Roux et al., 2021). Using metagenomics, low phage richness has previously been linked to Clustered Regularly Interspaced Short Palindromic Repeats (CRISPER)-Cas abundance (Meaden et al., 2022), suggesting a trade-off exists between this adaptive bacterial immune response and phage diversity. We screened the bacterial MAGs (n=2732) and identified a total of 18,840 antiviral defense genes on 1947 MAGs. Restriction-modification (n_genes_=8,705), Cas (n_genes_=4,483), abortive infection (n_genes_=1,365), and the Septu defense system (n_genes_=1,299) were the most commonly found defense types. MAGs contained up to 12 different antiviral defense system types (median number of defense types: 2) highlighting the general importance of antiviral defense for bacterial stream biofilm communities. Moreover, we found a significant relationship between viral diversity and the relative abundance of Cas-containing MAGs (Spearman’s rho = 0.49, p=0.0006, **Supplementary Figure S5**). Taken together, the diverse viral community and versatile antiviral defense arsenal in proglacial stream biofilms suggests a coevolutionary history of phages and biofilm-dwelling bacteria.

### Phage communities are coupled to bacterial host communities

**Figure 3:**
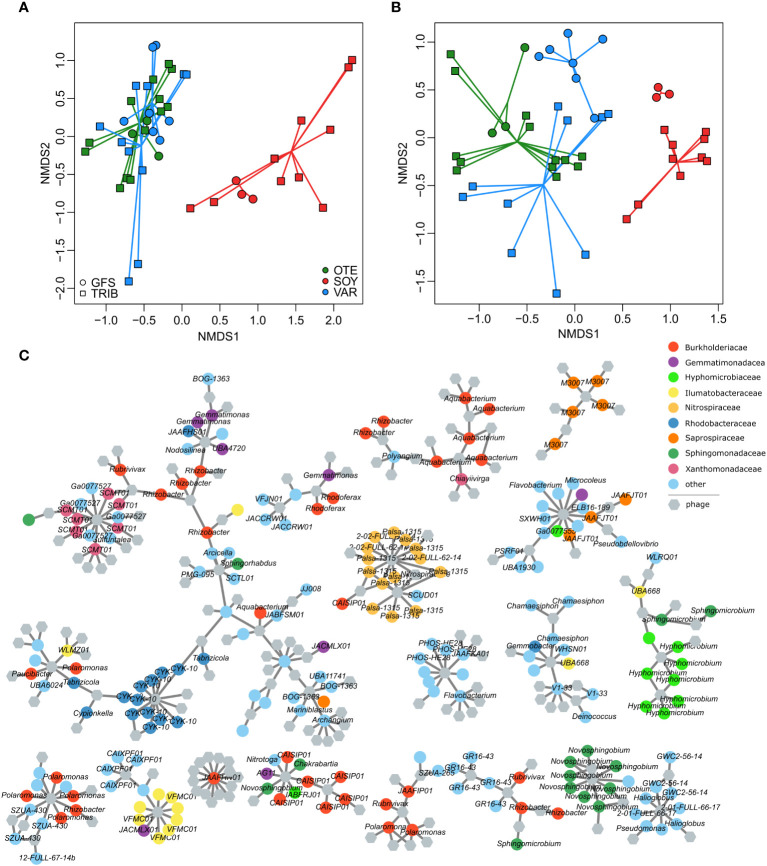
Phage-bacteria coupling. Non-metric multidimensional scaling ordinations based on vMAG relative abundance **(A)** and bacterial 16S rRNA gene ASV relative abundance **(B)** reveal the compositional dissimilarities among floodplains and stream types. This separation was more pronounced for the bacterial communities whereas viral communities were more similar (particularly samples of both GFS and GWS in OTE and VAR were similar). Resolving putative phage-host interactions (grey lines) among individual vMAGs (hexagons) and bacterial MAGs (colored circles) illustrate the generally high phage-host specificity (i.e. phages tend to interact with bacteria of similar taxonomy) **(C)**. The 17 largest sub-networks of the entire interaction network are shown.

Bacterial community composition based on Bray-Curtis similarity obtained from ASV relative abundance significantly differed between floodplains (PERMANOVA, R^2^ = 0.08, p<0.001) and stream types (PERMANOVA, R^2^ = 0.03, p<0.001), with a significant interaction between floodplain and stream type (PERMANOVA, R^2^ = 0.05, p<0.001). Additional variation can be explained by the spatial positioning of samples (e.g. distance to the glacier snout (Brandani et al., 2022), but substantial variation in the bacterial host community remains inexplicable. This highlights the large spatial heterogeneity among nearby streams in proglacial floodplains and the importance of stochastic community assembly processes in nascent ecosystems (Brandani et al., 2023). Moreover, bacterial communities from the Valsorey Glacier catchment were compositionally more distinct from communities of Otemma and Val Roseg, whereas particularly GWS sites of Otemma and Val Roseg were more similar (**Figure 3A**). This compositional similarity was mirrored by the viral community (**Figure 3B**), with significant differences between floodplains (PERMANOVA, R^2^ = 0.10, p<0.001) and stream types (PERMANOVA, R^2^ = 0.04, p<0.001), and a significant interaction term (PERMANOVA, R^2^ = 0.06, p<0.001). Superimposition of non-metric multidimensional scaling ordinations further substantiated the congruence between viral and bacterial communities (Procrustes correlation: 0.74, p=0.001, **Supplementary Figure S6**). The strength of these associations is comparable to earlier reports on the coupling of stream biofilm bacterial and viral communities (Bekliz et al., 2022) and suggests that host availability dominates over environmental conditions in shaping viral communities.

We used phage-host predictions (PHIST) to further resolve the coupling between bacterial and phage communities and predicted a total of 1435 phage-host interactions. Similar to other work (Luo et al., 2022), the majority of phage-host interactions (84.2%) involved a single phage-host pair and only six phages had more than 10 predicted interactions with different MAGs (maximum number of predicted interactions = 13). In line with generally high host specificity, vMAGs with multiple host interactions tended to interact with taxonomically similar MAGs (**Figure 3C**). For instance, 11 out of 13 interactions of a vMAG classified as *Lacusarxvirus* involved the alpha-proteobacterial CYK-10 genus. The other two interactions were predicted for MAGs classified as *Sphingomonas* (Alphaproteobacteria).

Phage-host interactions involving the microdiverse genera *Polaromonas*, *Rhizobacter*, *Rhodoferax*, *Methylotenera*, and *Massilia* were also dominated by single phage-host pairs (66 out of 110 interactions). Phages interacting with *Rhizobacter*, *Polaromonas*, and *Rhodoferax* also interacted with several other bacterial genera (**Supplementary Figure S7**). For instance, of the 38 vMAGs putatively interacting with *Rhizobacter*, 22 vMAGs exclusively interacted with *Rhizobacter* whereas 16 vMAGs also interacted with different bacterial genera. However, this was not consistent for all microdiverse bacterial clades: all of the 19 phages predicted to interact with the microdiverse genus *Methylotenera*, exclusively interacted with *Methylotenera* MAGs. Strikingly, only a single phage-host interaction was identified involving the microdiverse genus *Massilia*. Taken together, we discovered a continuum of potential phage host ranges but mostly found evidence for host specificity in proglacial streams.

While the limitations of *in silico* phage-host predictions need to be considered in this context (Brum and Sullivan, 2015; de Jonge et al., 2019), we report here a large number of putative phage-host interactions in stream biofilms, in line with reports from other extreme environments (Munson-McGee et al., 2018; Jarett et al., 2020). Moreover, the continuum of phage host range suggests that multiple eco-evolutionary processes shape bacteria-phage interactions in these communities (Weitz et al., 2013; de Jonge et al., 2019). On the one hand, trade-offs between virulence and host range (de Jonge et al., 2019) or resource limitation (Weitz et al., 2013) can explain narrow host ranges, whereas reduced dispersal and the dense packing of diverse bacteria have been associated with broader host ranges in biofilms (Hwang et al., 2023). In communities characterized by microdiversity, narrow host ranges may be beneficial for phages given the increased encounter probability with phylogenetically closely related members. Phages interacting with the microdiverse bacterial genus *Methylotenera* may, for instance, benefit from such narrow host range. On the other hand, broad host ranges may increase the likelihood of exchanging genetic material across distant bacterial populations. This may, for example, be the case for the microdiverse genus *Rhizobacter* in proglacial stream biofilms. We may thus hypothesize that beneficial effects of broad host ranges could contribute to the longer-term success of microdiverse bacterial clades. Microdiversity implies that phylogenetically closely related taxa occupy available niches. Typically, such closely related taxa are expected to compete for similar resources (Larkin and Martiny, 2017) and phage-mediated exchange of genetic material, including auxiliary metabolic genes, may alleviate competition among microdiverse clade members.

### Specific auxiliary metabolic genes are associated with microdiverse bacterial genera

In total, we detected 938 AMGs on 448 vMAGs, broadly reflecting the taxonomic composition of the entire vMAG community. In contrast to a recent analysis of Pearl River Estuary viromes (Luo et al., 2022), we did not detect differences in the number of AMGs between virulent and temperate phages. Specifically, 318 out of 1041 virulent vMAGs and 130 out of 411 temperate vMAGs harbored at least one AMG. When accounting for viral lifestyle distribution (i.e. 81.0% of vMAGs were virulent), both viral lifestyles represented a similar number of AMGs. Moreover, AMGs abundant among virulent phages were also abundant among temperate phages (Spearman’s rho = 0.72, p<0.0087).

AMGs encoded for a large functional diversity, with in total 166 different KEGG orthologues (KOs) present. Particularly numerous were AMGs encoding *dcm* (159 AMGs) which in prokaryotes can be involved in DNA methylation in restriction-modification systems (Pingoud et al., 2014). However, in phages, so-called orphan methyltransferases (i.e., without endonucleases of the prokaryotic restriction-modification system) can contribute to the protection of phage DNA against digestion (Heyerhoff et al., 2022). Other common AMGs were *queE, queD, queE, and folE* genes (137 AMG), involved in the synthesis of the deazapurine nucleoside preQ_O_, which also has been shown to protect viruses from restriction enzymes (Hutinet et al., 2019; Kieft et al., 2020). In contrast to pelagic systems (Kieft et al., 2021b; Heyerhoff et al., 2022) and despite the importance of cross-domain interactions in proglacial stream biofilms (Busi et al., 2022), however, AMGs encoding the core photosystem II proteins *psbA* and *psbD* were rare, with only 3 AMGs present across our entire dataset. The majority (34.4%) of AMGs encoded KOs involved in the metabolism of cofactors and vitamins including AMGs involved in folate biosynthesis, the metabolisms of nicotinate/nicotinamide, and porphyrine/chlorophyll. AMGs involved in amino acid metabolism (cysteine and methionine) accounted for another 20.7% of all AMGs. AMGs involved in carbohydrate metabolism (15.4%), glycan biosynthesis (8.4%), and sulfur metabolism (4.3%) were also commonly detected. Similar to marine sediments (Heyerhoff et al., 2022), *cysH*, which is involved in the synthesis of sulfite during assimilatory sulfate reduction, dominated AMGs involved in sulfur metabolism. *cysH* ranks among the globally conserved AMGs (Kieft et al., 2020) and has the potential to influence ecosystem-scale sulfur metabolism (Kieft et al., 2021a). This may be particularly relevant in GFS, where high turbidity and the absence of light fosters chemolithoautotrophic bacterial metabolisms. Chemolithoautotrophic bacteria can oxidize reduced sulfur to fix CO_2_ and to produce biomass. In the absence of photosynthetic organisms in GFS, chemolithoautotrophic carbon production may thus be an important ecosystem function, and phage AMGs may play a pivotal role.

**Figure 4:**
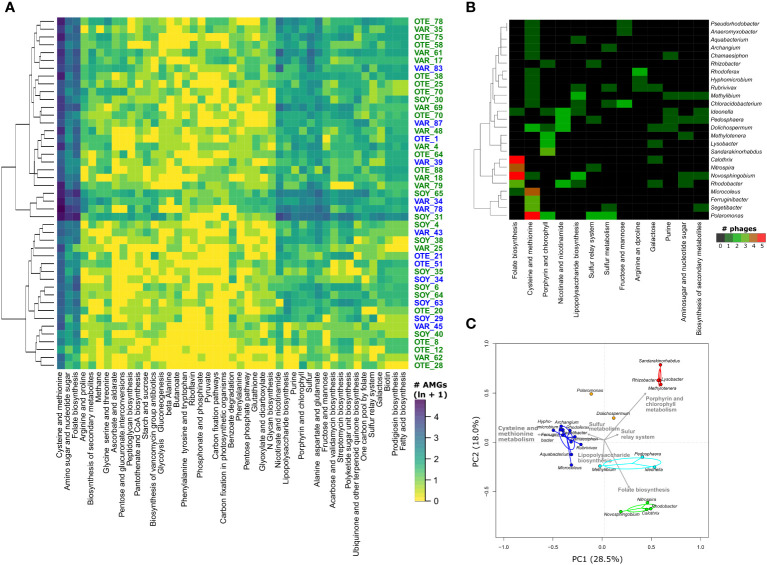
Auxiliary metabolic genes. A heatmap of the number of different AMGs found in the different samples highlights the prevalence of AMGs involved in the metabolism of cysteine and methionine, amino sugars and nucleotide sugar and folate biosynthesis **(A)**. The colors represent ln(x+1) transformed number of AMGs. Despite the pronounced compositional difference between GFS and GWS, no differences in AMG composition between GFS (blue sample IDs) and GWS (green sample IDs) were apparent. The dendrogram to the left of the heatmap shows the results of single-linkage clustering, hierarchically connecting samples based on similar AMG distributions. Coupling AMGs with phage-host interactions revealed AMGs shared by phages putatively infecting bacterial MAGs **(B)**. Shown are the 13 most common AMG-encoded pathways for 25 bacterial genera. Colors reflect the number of vMAGs. Considering all phage-host interactions and AMGs in a Principal Component Analysis, further highlighted the pronounced differences in phages carrying AMGs and infecting specific bacterial genera **(C)** Based on the number of AMGs on phages, different bacterial host genera (circles) cluster into distinct groups. Colored lines connect host genera to cluster centroids. Ellipses denote 95% confidence intervals for these clusters. Grey arrows denote KO pathways distinctive for the different clusters. For instance, the two microdiverse genera *Rhizobacter* and *Methylotenera* cluster with *Sandarakinorhabdus* and *Lysobacter* in terms of AMGs found on their respective phages. These AMGs are enriched in genes encoding porphyrin and chlorophyll metabolism. Note the distribution of microdiverse bacterial genera in different clusters and the absence of the microdiverse bacterial genus *Massilia*.

We did not detect significant differences in AMGs between viral communities from different floodplains or stream types (**Figure 4A**). Abundant AMG families, including the above-mentioned AMGs, tended to be present and abundant in most samples, whereas other AMG families were only sporadically present and often at low relative abundance. However, when coupling AMG with phage-host predictions (17.3% of AMGs were on vMAGs with a host prediction), we found that common and abundant AMGs were found on phages associated with specific bacterial community members (**Figure 4B**). For instance, 19 AMGs encoding folate biosynthesis were found on phages associated with 6 bacterial genera, including members of *Novosphingobium*, *Calothrix*, and *Rhodobacter*. Similarly, AMGs encoding for cysteine and methionine metabolism were particularly prevalent among vMAGs infecting members of the bacterial genera *Polaromonas*, *Microcoleus*, *Ferruginibacter*, and *Segetibacter*. Finally, using Principal Component Analysis with all AMGs for which phage-host predictions were available, we found distinct groups of bacterial genera to be host to phages with specific AMGs (**Figure 4C**). For example, the two microdiverse bacterial genera *Methylotenera* and *Rhizobacter*, together with *Lysobacter* and *Sandarakinorhabdus* formed a distinct group dominated by AMGs involved in porphyrine and chlorophyll metabolism. Another microdiverse bacterial genus, *Rhodoferax*, together with several other, non-microdiverse bacterial genera formed a distinct cluster based on AMGs involved in the metabolism of cysteine and methionine. AMGs involved in the biosynthesis of folate and lipopolysaccharides, explained clusters of bacterial genera involving members such as *Rhodobacter*, *Nitrospira*, *Novosphingobium*, and *Calothrix* as well as a cluster composed of *Methylibium*, *Pedosphaera*, and *Ideonella*. Interestingly, one of the most common and microdiverse bacterial genus in proglacial stream biofilms, *Polaromonas*, did not associate with any of the clusters. These findings highlight that similar AMGs may be found on phages associated with microdiverse and non-microdiverse clades.

Despite their compact genomes, phages regularly encode AMGs which can provide them a fitness advantage by augmenting or redirecting specific metabolic processes (Dion et al., 2020; Kieft et al., 2021b). This highlights the reciprocal benefits of phage-host interactions, which manifest during coevolution (Koskella and Brockhurst, 2014). The selective environmental constraints in proglacial streams have persisted over geological time scales, shaping microbial diversity and putatively influencing phage-bacteria coevolution. Host specificity of AMGs has been previously reported (Luo et al., 2022) and here we report specific AMGs linked with microdiverse (and non-microdiverse) clades in proglacial stream biofilms. Arguably, microdiverse clades and their phages have shared a long evolutionary time in a selective environment, refining the AMGs library and putatively contributing to the eco-evolutionary success of both, the phages and their prokaryotic hosts. Microdiversity is an intrinsic property of many microbial communities and horizontal gene transfer can generate microdiversity (Larkin and Martiny, 2017). Phage-mediated HGT transfer may therefore contribute to microdiversification and narrow host range and AMG specificity suggests that in proglacial stream biofilms, virus-host interactions may foster ‘Maestro Microbes’ (Larkin and Martiny, 2017), which occupy niches by optimizing specific traits (in contrast to ‘Renaissance Microbes’ which acquire new traits (Larkin and Martiny, 2017).

## Conclusions

Generally, little is known about viral biodiversity in extreme environments, particularly however in streams and rivers draining mountain regions (Payne et al., 2020; Bekliz et al., 2022; Busi et al., 2022). Here, we expand the current knowledge of phage diversity and its role in shaping microbial communities in streams and rivers. Biofilms are thought to represent a physical barrier for viruses (Abedon, 2011), and our work unravels viral communities, which are shaped by their bacterial host community composition. Extreme environmental conditions in proglacial streams lead to strong selective sorting of these host communities, ultimately resulting in communities dominated by few but well-adapted and microdiverse clades. Contrary to our expectation of generally narrow phage-host ranges in communities dominated by microdiverse bacterial clades, we found a continuum of host ranges, which we attribute to different eco-evolutionary dynamics, putatively associated with resource limitation in proglacial streams (explaining narrow host ranges) and the biofilm mode of life (putatively explaining broader host ranges). Phages infecting members of different microdiverse bacterial genera carry AMGs, which appear to be distinct and specific. Future work may further elucidate the role of non-microdiverse clades, which share similar AMGs and assess to which degree the coevolution between phages and their hosts shapes the AMG repertoire. This may be particularly relevant in extreme environments, where stark selective processes shape communities. On the other hand, microdiversity is a hallmark of many microbial communities, and beneficial phages-host interactions may play important roles in generating and maintaining microdiversity.

## Data availability statement

The datasets presented in this study can be found in online repositories. The names of the repository/repositories and accession number(s) can be found below: https://www.ncbi.nlm.nih.gov/, PRJNA808857. **Supplementary Table S1** lists individual BioSample accession numbers. Raw bioinformatic output is available at Figshare under doi 10.6084/m9.figshare.24511972.

## Author contributions

HP: Conceptualization, Formal Analysis, Investigation, Methodology, Visualization, Writing – original draft. GM: Conceptualization, Data curation, Formal Analysis, Investigation, Methodology, Writing – original draft. SB: Conceptualization, Data curation, Formal Analysis, Investigation, Methodology, Writing – original draft. TB: Conceptualization, Funding acquisition, Project administration, Resources, Supervision, Writing – original draft.
