# Supplementary material for: The role of phages for microdiverse bacterial communities in proglacial stream biofilms
Source: Front Microbiomes. 2024 Jan 15;2:1279550. doi: 10.3389/frmbi.2023.1279550 (PMC12993515; doi:10.3389/frmbi.2023.1279550)
Supplement: Supplementary file 1 [file DataSheet_1.pdf]

Supplementary Material

for

The role of phages for microdiverse bacterial communities in proglacial stream biofilms

Hannes Peter[1,*], Gregoire Michoud[1], Susheel Bhanu Busi[2,3], Tom J. Battin[1]

Supplementary Table S1 Sample specific accession numbers as well as geographic and sampling information.

ID	BioSample Accession Numbers	# reads mapped to vMAGs	percentage of reads mapped to vMAGS	x coordinate (LV95)	y coordinate (LV95)	z coordinate (LV95)	glacier floodplain	sampling period *	stream_ type
OTE_1	SAMN26116630	1771835	0.89%	2597650	1086739	2448.381	OTE	early	GFS
OTE_12	SAMN26116634	1005577	0.66%	2598027	1087090	2454.726	OTE	early	GWS
OTE_14	SAMN26116636	649657	0.31%	2598048	1087122	2455.672	OTE	early	GWS
OTE_20	SAMN26116643	2374943	1.39%	2598155	1087183	2456.464	OTE	early	GWS
OTE_21	SAMN26116644	922132	0.55%	2598202	1087149	2455.947	OTE	early	GFS
OTE_25	SAMN26116648	904898	0.53%	2598304	1087274	2460.523	OTE	early	GWS
OTE_28	SAMN26116650	1294831	1.21%	2598462	1087335	2465.624	OTE	early	GWS
OTE_38	SAMN26116661	1344774	0.62%	2598313	1087116	2457.535	OTE	early	GWS
OTE_51	SAMN26116675	786522	0.38%	2597650	1086739	2448.381	OTE	late	GFS
OTE_58	SAMN26116682	1232704	0.52%	2597722	1086860	2450.472	OTE	late	GWS
OTE_64	SAMN26116689	658444	0.35%	2598048	1087122	2455.672	OTE	late	GWS
OTE_70	SAMN26116696	1107108	0.69%	2598155	1087183	2456.464	OTE	late	GWS
OTE_70b	SAMN26116696	1382207	0.82%	2598155	1087183	2456.464	OTE	late	GWS
OTE_75	SAMN26116701	1162445	0.67%	2598304	1087274	2460.523	OTE	late	GWS
OTE_78	SAMN26116704	1882523	0.92%	2598462	1087335	2465.624	OTE	late	GWS
OTE_8	SAMN26116706	2032752	1.09%	2597722	1086860	2450.472	OTE	early	GWS
OTE_88	SAMN26116715	1920800	1.06%	2598313	1087116	2457.535	OTE	late	GWS
SOY_1	SAMN26116727	802690	0.36%	2585985	1085339	2390.802	SOY	early	GWS
SOY_29	SAMN26116748	865035	0.40%	2585923	1085465	2388.982	SOY	early	GFS
SOY_30	SAMN26116750	948654	0.56%	2585937	1085507	2388.176	SOY	early	GWS
SOY_31	SAMN26116751	2067115	1.18%	2585958	1085609	2394.884	SOY	early	GWS
SOY_34	SAMN26116754	928936	0.44%	2585842	1085588	2382.816	SOY	early	GFS
SOY_35	SAMN26116755	759679	0.39%	2585985	1085339	2390.802	SOY	late	GWS
SOY_38	SAMN26116758	2163154	1.50%	2586087	1085262	2390.137	SOY	late	GWS
SOY_4	SAMN26116760	1671432	1.12%	2586087	1085262	2390.137	SOY	early	GWS
SOY_40	SAMN26116761	767469	0.60%	2586111	1085247	2390.063	SOY	late	GWS
SOY_6	SAMN26116782	936379	0.45%	2586111	1085247	2390.063	SOY	early	GWS
SOY_63	SAMN26116786	909883	0.46%	2585923	1085465	2388.982	SOY	late	GFS
SOY_64	SAMN26116787	1206978	0.64%	2585937	1085507	2388.176	SOY	late	GWS
SOY_65	SAMN26116788	2050472	1.19%	2585958	1085609	2394.884	SOY	late	GWS
VAR_17	SAMN26116803	2054307	1.24%	2786094	1142795	2112.052	VAR	early	GWS
VAR_18	SAMN26116804	1325985	0.67%	2786151	1142749	2117.99	VAR	early	GWS
VAR_25	SAMN26116812	1111427	0.65%	2786207	1142644	2130.186	VAR	early	GWS
VAR_34	SAMN26116822	3195028	1.85%	2786460	1142342	2203.038	VAR	early	GFS
VAR_35	SAMN26116823	1451765	0.69%	2786477	1142345	2205.934	VAR	early	GWS
VAR_39	SAMN26116827	1050564	0.52%	2786496	1142288	2216.633	VAR	early	GFS
VAR_4	SAMN26116828	2380608	1.27%	2785955	1143054	2095.481	VAR	early	GWS
VAR_43	SAMN26116832	967146	0.61%	2786706	1142166	2261.76	VAR	early	GFS
VAR_45	SAMN26116834	898812	0.54%	2785894	1143174	2089.271	VAR	late	GFS
VAR_48	SAMN26116837	2205865	1.27%	2785954	1143054	2096.552	VAR	late	GWS
VAR_61	SAMN26116852	1462696	0.76%	2786095	1142794	2113.062	VAR	late	GWS
VAR_62	SAMN26116853	859526	0.39%	2786150	1142749	2118.937	VAR	late	GWS
VAR_69	SAMN26116860	2695038	1.28%	2786208	1142643	2131.163	VAR	late	GWS
VAR_78	SAMN26116870	2932028	1.37%	2786460	1142342	2203.666	VAR	late	GFS
VAR_79	SAMN26116871	1978714	0.95%	2786477	1142344	2207.087	VAR	late	GWS
VAR_83	SAMN26116876	2560010	1.14%	2786498	1142290	2219.346	VAR	late	GFS
VAR_87	SAMN26116880	2022794	1.14%	2786707	1142168	2262.66	VAR	late	GFS

* sampling periods: early: June/July late: August/September

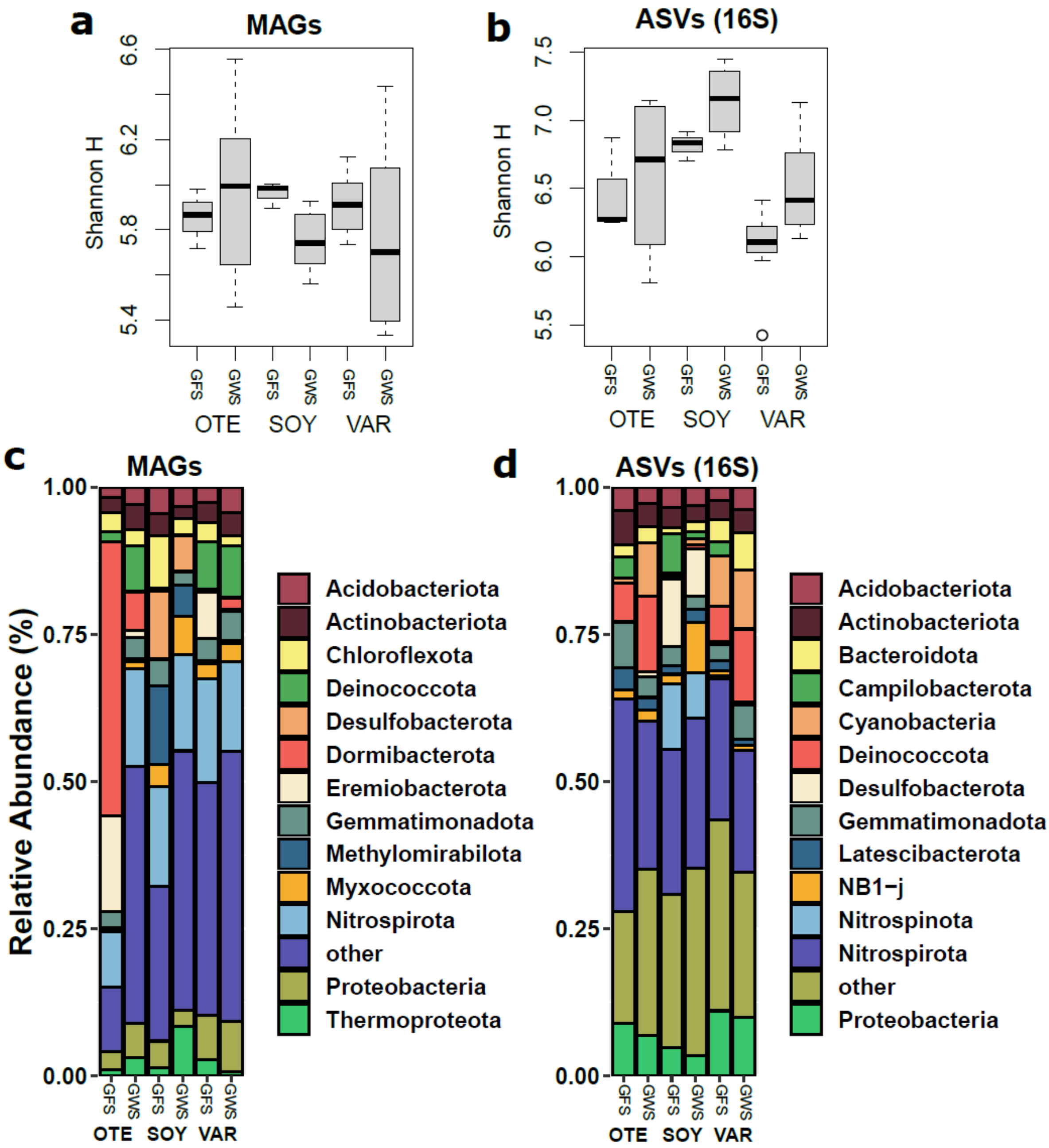

Supplementary Figure S1 Comparison between Metagenome-Assembled Genome (MAG) and Amplicon Sequence Variants (ASVs, 16S rRNA gene). Shown are diversity estimates (Shannon H) for samples obtained from the different floodplains and stream types for MAGs (a) and ASVs (b), respectively. Panel c and d show taxonomic composition at phylum level for the 14 most abundant (on average across all samples) taxonomic bins for MAGs (c) and ASVs (d).

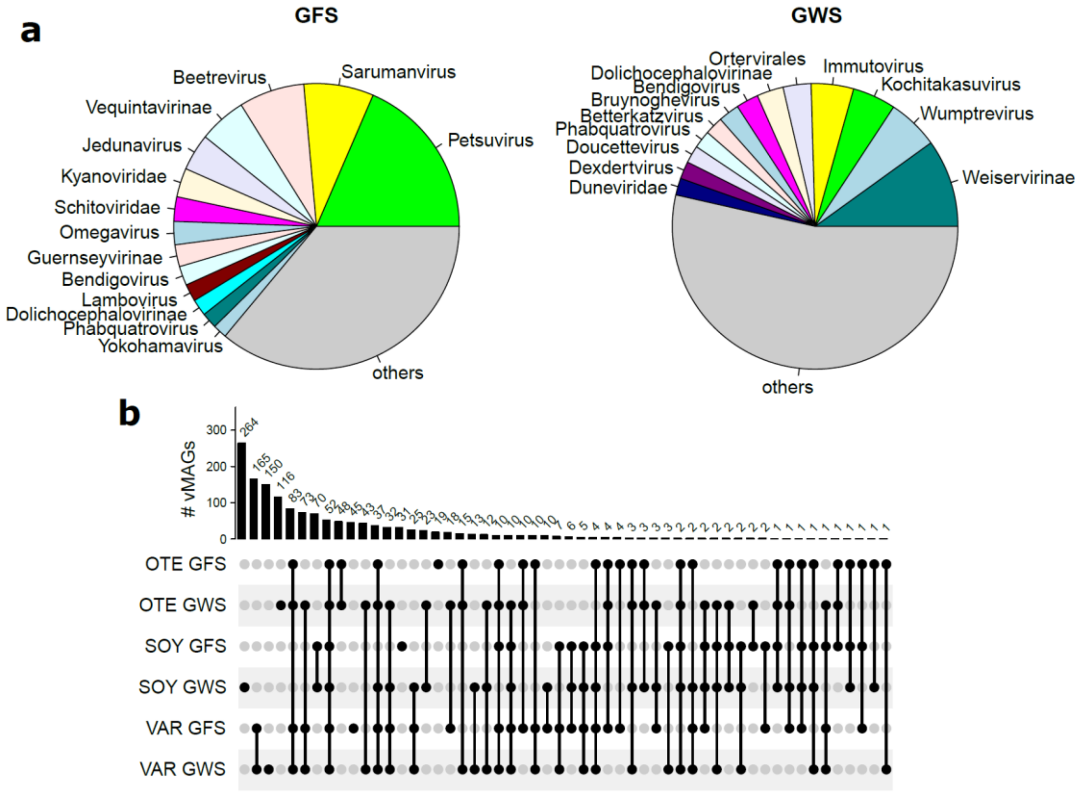

Supplementary Figure S2 Abundance and prevalence of vMAGs across proglacial streams. DESeq2 identified 697 vMAGs either significantly enriched in GFS (n=139 vMAGs) or in GWS (n=558 vMAGs) (panel a). The pie charts show the taxonomic composition of GWS and GFS-enriched vMAG, respectively. The upset plot shown in panel b provides information regarding the number of vMAGs found across proglacial stream types. Lines connecting two or more categories represent the shared number of vMAGs. For example, 264 vMAGs were exclusively detected in GWS of SOY and 165 vMAGs were found in both GFS and GWS of VAR but in no other stream.

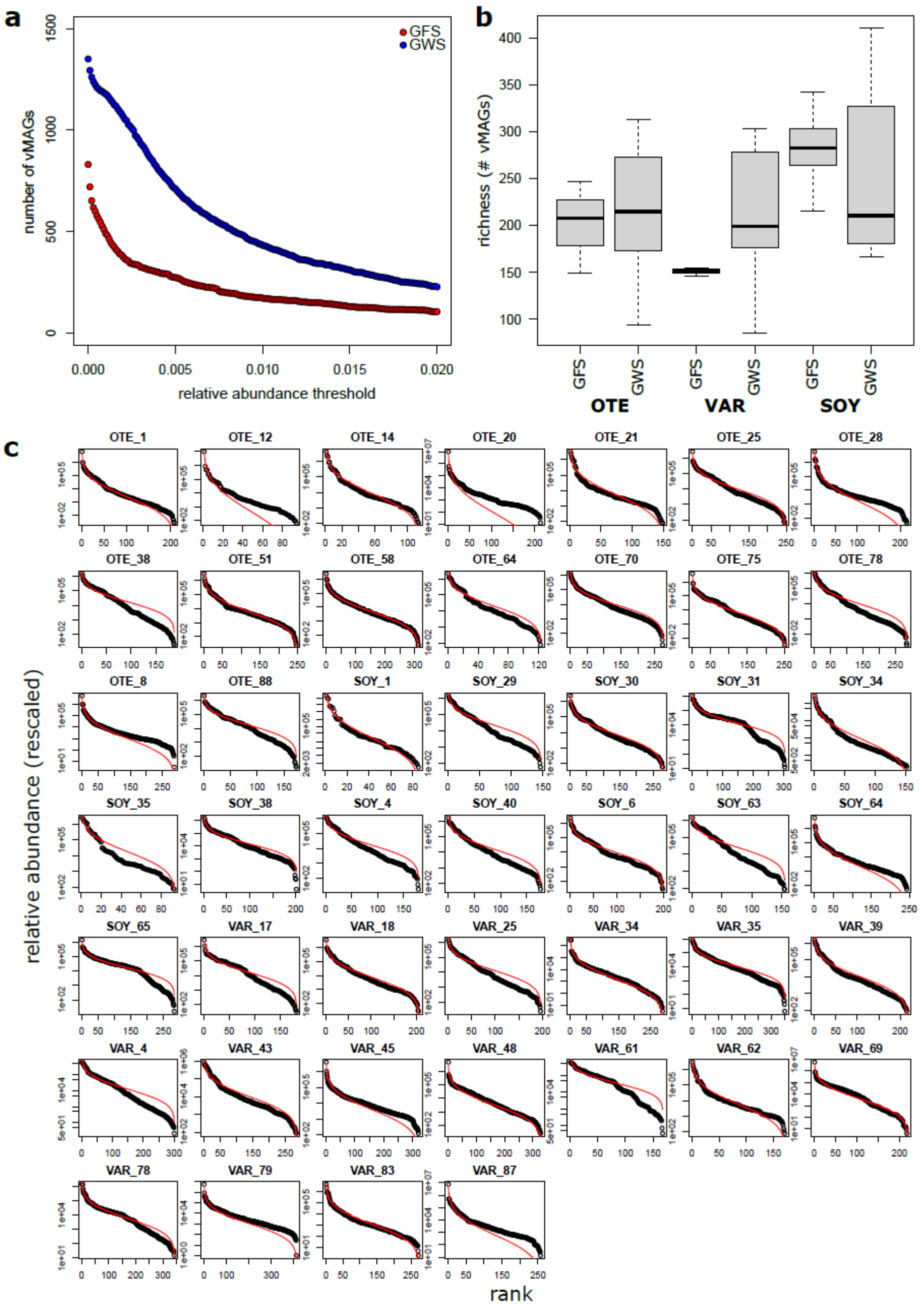

Supplementary Figure S3 Low abundant vMAGs in proglacial stream biofilms. We detected a large number of vMAGs with low relative abundance, particularly in GFS. Relative abundance of vMAGs was calculated as the normalized number of reads mapped to vMAGs in each sample. We consider vMAGs as being present in a sample if at least 5% of their genome was covered by reads (values lower than 5% were set to zero). Panel a provides a sensitivity analysis of how the number of vMAGs changes along an increasing threshold of relative abundance. Note that while Panel b shows vMAG richness across floodplains and stream types. There were no significant differences in richness among these categories. Panel c displays empirical rank-abundance distributions (black symbols) for all samples. For this, vMAGs are ranked from most to least abundant in each sample. Relative abundance was rescaled such that the lowest relative abundance in each sample equaled one. Using function radfit implemented in R, rank abundance models were fit for each sample and log-normal model fits (red lines) often approximated the empirical rank abundance distributions well.

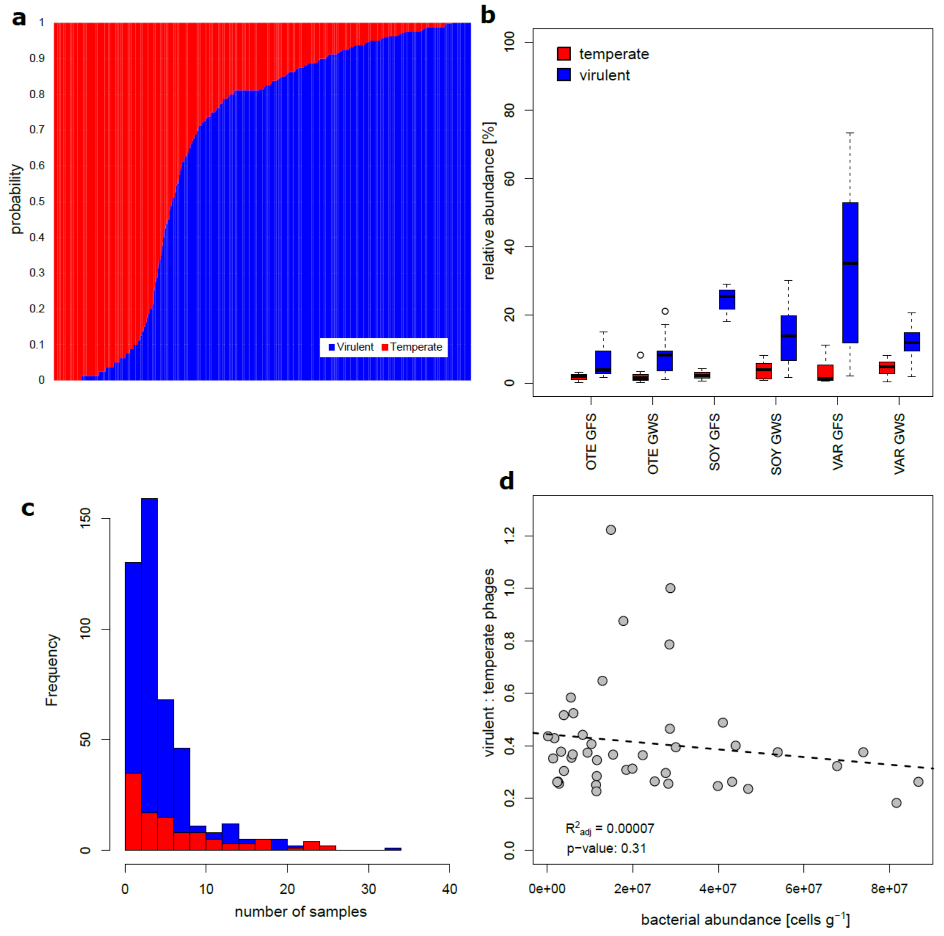

Supplementary Figure S4 Temperate and virulent phage lifestyles. Viral lifestyles were assigned to 582 complete vMAGs. Panel a displays bacphlip assignment confidence for all 582 vMAGs. Panel b shows the contribution to relative abundance of temperate and virulent complete vMAGs across proglacial floodplains and stream types. Panel c displays the frequency distribution of prevalence (i.e. the number of samples a vMAG was found) for temperate and virulent complete vMAGs. Colors are the same as in panel b. Panel d shows the (non-significant) relationship between bacterial cell numbers in proglacial stream sediment samples and the ratio of virulent : temperate vMAGs.

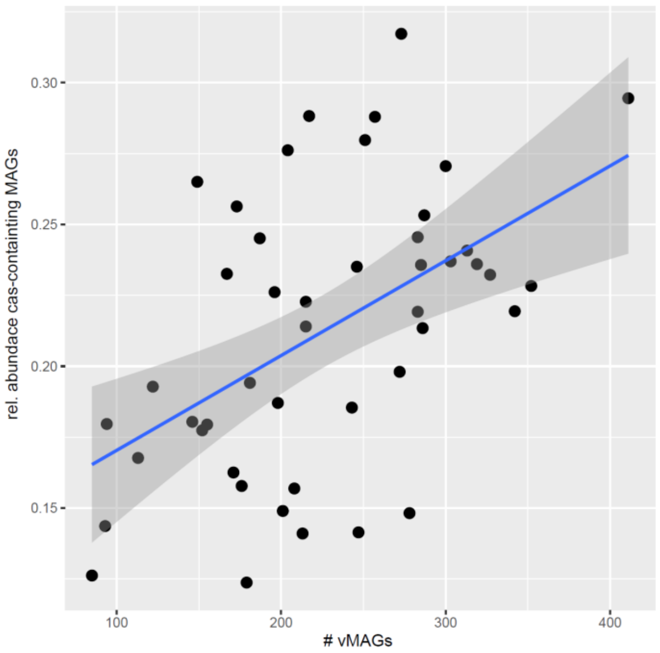

Supplementary Figure S5 In line with previous work (Meaden et al. 2022), we observed a significant relationship between the number of vMAGs detected in each sample and the relative abundance of cas-containing MAGs, suggesting tradeoffs between bacterial response and phage diversity.

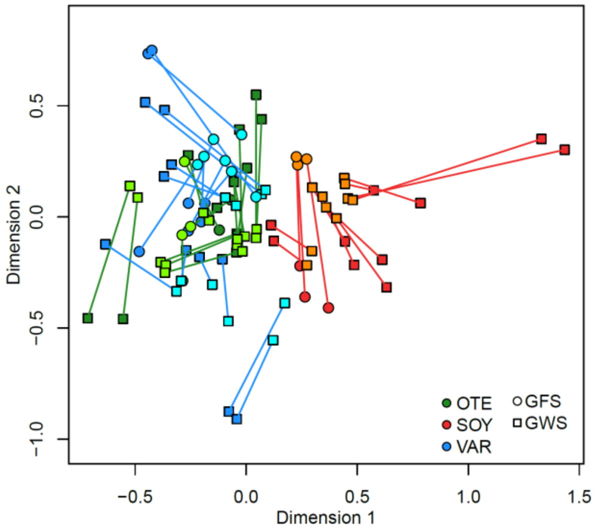

Supplementary Figure S6 Procrustes Analysis between phage and bacterial community. The superpositioning of two NMDS ordinations (Figure 3, main text) demonstrates the significant coupling between phage and bacterial community similarity (Bray Curtis). Phage (light colors) and bacterial (darker colors) community composition were significantly correlated across the different stream types and floodplains. Note that the strength of this coupling is similar for all community pairs (indicated by the length of the lines).

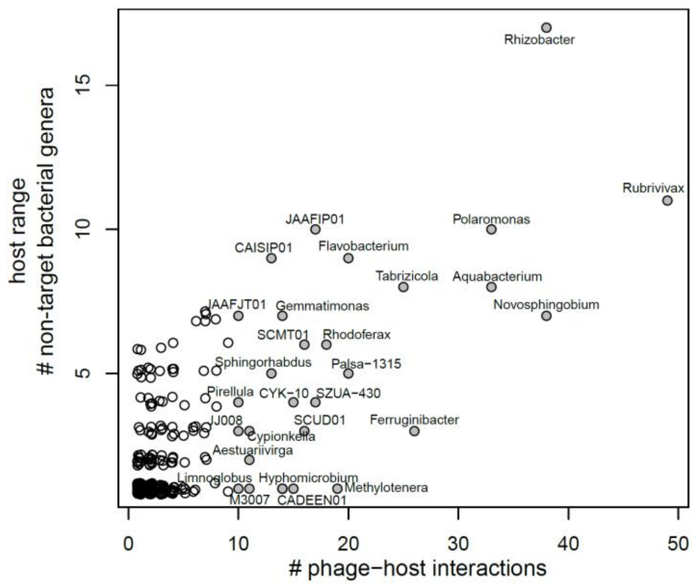

Supplementary Figure S7 Phage host interactions and host range. Shown are, for each bacterial genus (i.e. target genus), the number of phage-host interactions versus phage host range (i.e. the total number of bacterial genera that target-genus phages interact with). Most phages interacted with a single or only few bacterial hosts (open circles). The taxonomy for target genera with multiple phage host-interactions (filled circles) is displayed. Note that microdiverse genera, such as *Polaromonas* and *Rhizobacter* have many predicted phages, which also infect several other bacterial genera.
